# High health gain patients with asthma: a cross-sectional study analysing national Scottish data sets

**DOI:** 10.1038/s41533-018-0094-6

**Published:** 2018-07-19

**Authors:** Mome Mukherjee, Bright I. Nwaru, Ireneous Soyiri, Ian Grant, Aziz Sheikh

**Affiliations:** 1 Asthma UK Centre for Applied Research, Centre for Medical Informatics, Usher Institute of Population Health Sciences and Informatics, The University of Edinburgh, Edinburgh, UK; 2 Krefting Research Centre, Institute of Medicine, University of Gothenburg, Gothenburg, Sweden; 3 Wallenberg Centre for Molecular and Translational Medicine, Institute of Medicine, University of Gothenburg, Gothenburg, Sweden; 4 ScotPHO Collaboration, Information Services Division (ISD), NHS National Services Scotland, Edinburgh, UK

## Abstract

Studies have shown that a small proportion of patients have particularly high needs and are responsible for disproportionally high disease burden. Estimates suggest that 2–5% of patients are high users of healthcare for their health gain. Such patients in Scotland are referred to as high health gain (HHG) patients. We wanted to investigate if there were HHG individuals with asthma in Scotland. We analysed data from the Scottish Health Survey (2010–11), and primary and National Health Survey (NHS) secondary healthcare and administrative data sets (2011–12). In all, 1,379,690 (26.0%) and 836,135 (15.8%) people reported to have ever had and currently have symptoms suggestive of asthma, respectively; 369,868 (7.0%) people reported current symptomatic clinician-diagnosed asthma. 310,050 (5.6%) people had clinician-reported-diagnosed asthma; there were 289,120 nurse consultations, 215,610 GP consultations, 9235 accident and emergency visits (0.2% people), 8263 ambulance conveyances (0.2% people), 7744 inpatient episodes (0.1% people), 3600 disability allowance claims (0.1% people), 187 intensive care unit (ICU) episodes and 94 deaths from asthma. From our study a maximum of about 9.4% of asthma patients (*n* = 29,145), which is 0.5% of the Scottish population, and from the National Review of Asthma Deaths’ estimate (10% hospitalised), a minimum of nine people had severe asthma attacks that needed acute hospital attendance/admission. We found that although a high proportion of the Scottish population had symptoms suggestive of asthma and clinician diagnosed asthma, only a small proportion of asthma patients experienced exacerbations that were severe enough to warrant hospital attendance/admission in any given year. Developing risk prediction models to identify these HHG patients has the potential to both improve health outcomes while substantially reducing healthcare expenditure.

## Asthma: Identifying high risk patients for early intervention

Insights from a detailed analysis of asthma severity in the Scottish population could help researchers develop a tool to identify high risk patients. A small percentage of asthma patients are classed as ‘high health gain’ (HHG) patients — those with severe symptoms who use healthcare services more regularly than others. However, national data on these difficult-to-identify HHG cases have not been explored. Mome Mukerjee and co-workers at the University of Edinburgh, UK, analyzed two Scottish national databases covering primary and secondary care data gathered over two years. 5.6 per cent of the Scottish population have clinician-diagnosed asthma, and only a small percentage (0.5) would be classed as HHG. Further stratified risk analysis at population level could inform future intervention strategies, including creating tools to identify patients who require significant intervention to prevent hospitalisation or death.

## Introduction

Asthma is now one of the most common long-term conditions in economically-developed countries, with the prevalence being particularly high in English-speaking nations.^1^ There is also a growing body of work showing that asthma is responsible for considerable morbidity and, in some cases, mortality.^2^

While there are a number of investigations on the epidemiology and healthcare utilisation of asthma in specialist clinical settings and some using more population-based approaches,^3–9^ there have been relatively few attempts to provide a national overview of asthma and its impact.^2^ Such analyses are potentially important to support health policy and planning, as well as inform deliberations on whether there are subsets of the asthma population that are particularly at high risk of poor outcomes and thus need focussed care. This subset of the population is also sometimes known as ‘high-need, high-cost’ (HNHC) patients and in Scotland as ‘high risk individuals’ (HRI) or ‘high health gain’ (HHG) individuals.^10–16^

Building on our recent work studying asthma in the UK,^2^ we sought to undertake a more detailed analysis of asthma in Scotland, taking advantage of the considerable data assets available, in order to provide the first detailed national profile of asthma in Europe. This work will enable us to ascertain if there is a HHG group of individuals with asthma.

## Results

### Prevalence

In 2010–11, an estimated 26.0% (95% CI 21.7–30.4; equivalent to 1,379,690 people, 95% CI 1,148,779–1,610,601) of the Scottish population reported to ever had experienced symptoms of asthma in their lifetime; 15.8% (95% CI: 12.4–19.2; equivalent to 836,135 people, 95% CI 655,872–1,016,398) reported to have experienced symptoms of asthma in the last 12 months preceding the survey; 13.6% (95% CI 10.5–16.7; equivalent to 746,542 people, 95% CI 554,883–884,271) reported to have had clinician-diagnosed asthma in their lifetime; 9.9% (95% CI 7.3–12.5; equivalent to 524,883 people, 95% CI 385,831–663,935) reported to have had clinician-diagnosed symptomatic asthma in the last 12 months; and 7.0% (95% CI 4.8–9.2; equivalent to 369,868 people, 95% CI 251,933–487,802) reported to have had clinician-diagnosed-and-treated asthma in the last 12 months. In 2011–12, an estimated 5.6% (95% CI 5.5–5.7; equivalent to 310,050 people, 95% CI 305,182–314,918) had clinician-diagnosed asthma and 6.0% (95% CI 6.0–6.0; equivalent to 319,091 people, 95% CI 319,091–319,091) had clinician-diagnosed-and-treated asthma.

### Healthcare and societal care utilisation

In 2011–12, there were 289,120 nurse consultations (95% CI 284,422–293,818); 215,610 GP consultations (95% CI 211,563–219,657); 9235 A&E attendances (95% CI 8358–10,113); 8263 ambulance conveyances; 7744 inpatient episodes; 4575 out of hour calls; 3600 claimed DLA; there were 187 ICU episodes and 94 deaths.

Since this was a cross-sectional study, we could not ascertain what proportion of asthma patients contributed to the healthcare use. However, with the conservative assumption of one event or service use to one person only, and using the lowest denominator (i.e., 310,050 as the number of people with clinician-diagnosed asthma reported by GPs), then we can estimate that 93.2% (95% CI 91.7–94.8) had nurse consultations; 69.5% had GP (95% CI 68.2–70.8); 1.5% called out-of-hours service (95% CI 1.4–1.5); 3.0% attended A&E (95% CI 2.7–3.3); 2.7% used an ambulance service (95% CI 2.6–2.7); 2.5% had a hospital admission (95% CI 2.4–2.6); 1.2% claimed DLA (95% CI 1.1–1.2); 0.1% were admitted in an ICU (95% CI 0.1–0.1); and 0.03% died with asthma as an underlying reason (95% CI 0.02–0.04). This implies that in Scotland there was a maximum of about 9.4% of asthma patients (sum of 3% A&E attendances, 2.7% ambulance conveyances, 2.5% hospital admission, 1.2% DLA claims, 0.1% ICU, 0.03% deaths), equivalent to 29,145 people, which is 0.5% of the population, who had severe asthma, which required resource intensive ambulance services, acute hospital services (i.e., A&E/hospitalisation/ICU admission), costly DLA benefits or led to death.

**Fig. 1:**
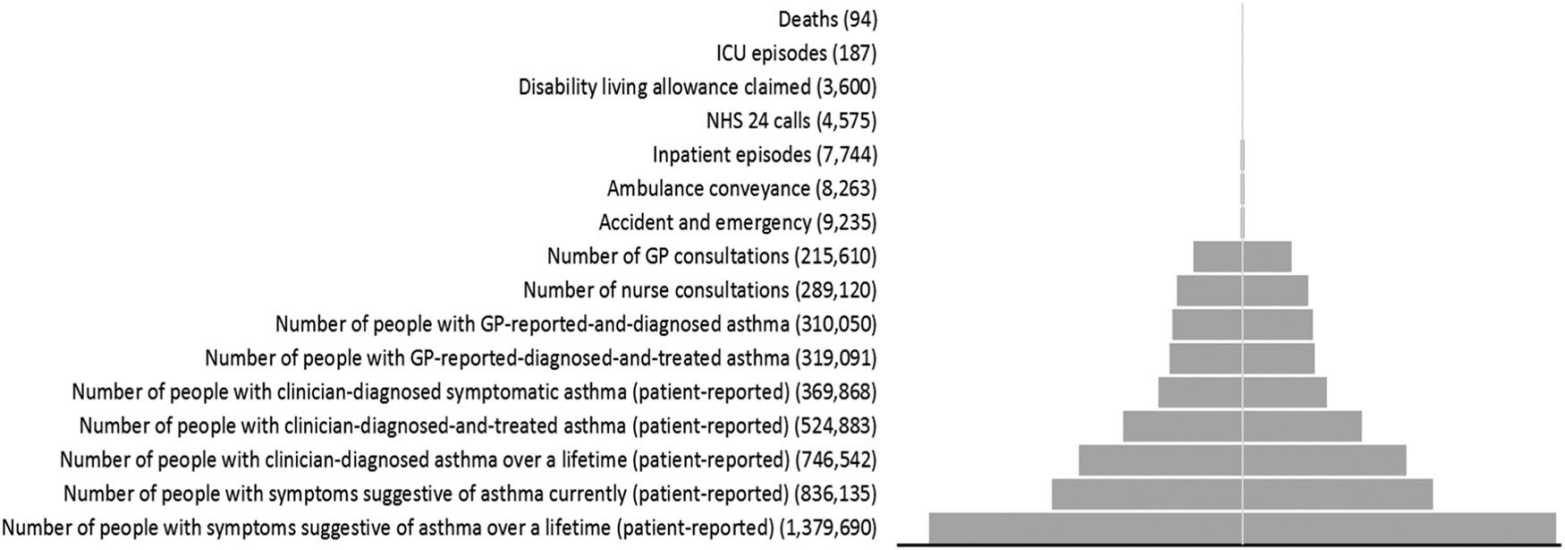
Asthma population profile in Scotland in 2011–12 (patient reported 2010–2011): asthma prevalence, healthcare utilisation and outcomes

The asthma population profile, describing asthma prevalence, healthcare utilisation and outcomes, including mortality, is illustrated using a pyramid graph below (Fig. 1). The stack of the pyramid is depicting the severity of the disease at population level. The bottom of the pyramid is showing the number of people who had ever experienced symptoms of asthma in their lifetime (*n* = 1,379,690), whereas the tip is showing the number of people who eventually died due to asthma as the underlying reason (*n* = 94).

## Discussion

### Statement of principal findings

This national level profile of asthma epidemiology and health and social care utilisation in Scotland has found that even though the annual prevalence of symptoms suggestive of asthma (15.8% of the Scottish population) or of clinician-diagnosed asthma itself (5.6% of the Scottish population) is high in Scotland, only a small minority of patients (0.5% of the Scottish population) experience events severe enough to lead to emergency hospital attendance/admission, with far fewer still who experience ICU admission or have a fatal episode in any given year. This suggests that there may be potential to risk-stratify at a population level and develop cost-effective interventions in this high risk group.

### Strengths

This study provides the first detailed national picture of asthma in Scotland using population representative survey data and routine data from a national health system and administrative data, with coverage from cradle-to-grave. The estimates produced in this exercise are slightly higher than our previous exercise.^2^ Are these estimates better? Our previous exercise was to compare nations across the UK. Hence, we had used European Standard Population (ESP) 2013 as the reference population when we age standardised. But when we are focussing on Scotland and not comparing to elsewhere, using the population estimate of Scotland (SMYEP) as the reference is a logical choice. If we compare ESP and SMYEP, we will find that: (a) population distribution by gender in ESP is identical, whereas in SMYEP it varies (online Appendix 2); and (b) SMYEP had less young population and higher older population compared to ESP. Thus, by using SMYEP split by 5-year age-groups and gender as the reference population, the estimates are not only different, but also higher. The reference population used to produce the estimates is appropriate for this particular study, hence these estimates are more appropriate for this purpose.

### Limitations

We did not have cohort data to investigate if there is a small group of asthma patients who have high-need and thus use healthcare services more. In the absence of cohort data, the layout of this cross-sectional study amply suggests that there is a tiny group of asthma patients who have severe asthma and use resource intensive or costly services. Since this was not a cohort study, one step of the pyramid did not necessarily contribute to the next. Our estimates are based on conservative assumptions. Thus, we presume that the proportion of asthma patients who had severity or fatality are even smaller than we estimated here (9.4%). Nevertheless, this study lends us a hypothesis of the proportion of HNHC/HRI/HHG asthma patients, i.e., a maximum of 9.4% of asthma patients could be HNHC/HRI/HHG. Some data gaps we found were unavailability of out-of-hours GP services data, lack of diagnosis data in outpatient clinics, people who got Xolair (drug used to treat severe asthma), people who used private medical services for their asthma. Although we had good coverage of data, there were two NHS health boards, which did not contribute data to A&E2 and thus were excluded in this analysis.

### Interpretation in the light of the previous literature

Of the 16 outcome measures reported here, nine outcomes had the same *values as* in our previous exercise,^2^ since either they were *real absolute numbers* and cannot/should not be adjusted to population level, example number of calls to NHS 24, number of ambulance trips, number of day-case/inpatient episodes, number of deaths, or they were already adjusted to the respective database population, example GP consultations, nurse consultations and annual prevalence of clinician-reported-and-diagnosed asthma, all from Practice Team Information (PTI) primary care database, or the data did not have age-sex distribution and hence could not be adjusted, example annual prevalence of clinician-reported-diagnosed-and-treated asthma from Quality and Outcomes Framework (QOF), Disability living allowance (DLA). The other seven estimates generated from using SMYEP are more appropriate when looking into Scotland alone. These seven *new* estimates were for five prevalence estimates from Scottish Health Survey, A&E and ICU. For the five prevalence estimates from Scottish Health Survey, the number of respondents who had said yes to the question (*n*), number of respondents (*N*), ASR (95% CI), were provided for Scotland in the previous exercise.^2^ The age and gender adjusted rates were different when SMYEP was used, and thus the *estimated number* reported in this manuscript. A&E estimates for the two HBs, which did not submit patient level data were generated adjusting to the respective HB’s estimated population and were estimated for Scotland using SMYEP. We did not have the number of children under 15 in adult ICUs (from ICNARC) for England, Wales and Northern Ireland. But we had small number of children who were 15 and older in paediatric ICUs in PICANet and children under 15 in adult ICUs in SICSAG for Scotland. These small numbers had to be excluded in our earlier exercise to follow the same principle of inclusion across the four nations.^2^ Since this manuscript is about Scotland specific estimate, we could use these small numbers by combining data from PICANet and SICSAG.

We found that while 310,050 people who were diagnosed for asthma by clinicians (5.6% people (95% CI: 5.5–5.7)), 319,091 people (6.0% people (95% CI: 6.0–6.0)) had clinician-diagnosed-and-treated asthma as per the financial incentive based QOF register. Perhaps there was an issue of possible over-diagnosis or over-labelling, as was found in studies on children and adults in the Netherlands and Canada, respectively.^17,18^ There is likely to be scope for diagnostic clarification in the population with asthma symptoms.

This work shows that only a small proportion (0.5%) of the Scottish population end up with serious asthma attacks or deaths. We know from our previous exercise that of the approximately £92.2 million public expenditure in Scotland for asthma,^2^ about £54.5 m (59.1%) was spent on community prescriptions, £14.8 m (16%) on DLA, £8.6 m (9.3%) on GP consultations, £6.3 m (6.9%) on inpatient episodes, £4.0 m (4.4%) on nurse consultations, £2.4 m (2.6%) on ambulance conveyance, £0.9 m (1.0%) on A&E, £0.5 m (0.5%) on ICU and £0.1 m (0.1%) on out-of-hour calls.^2^ Due to data constraints in that exercise we could not calculate cost per patient for use of each of those healthcare services, which would have helped us understand resource use of a healthcare service in monetary units at a person level. However, using our conservative assumption above, we can estimate that 1.2% people with clinician-diagnosed asthma who claimed DLA, cost the economy £14.8 m; 2.5% who had a hospital admission for asthma cost the economy at least £6.3 m; and 0.1% who were admitted in an ICU had cost the economy at least £0.5 m.

In the UK-wide review of asthma deaths, NRAD had found that of the 195 deaths for asthma, 21% had been to A&E and 10% were hospitalised for their asthma in the 12 months prior to their deaths. These UK-wide percentages are much higher than our conservative estimates for Scotland. Yet, if we apply the NRAD percentages to the Scottish asthma deaths, we reckon of the 94 people who died due to asthma, 20 might had been to A&E and 9 were hospitalised in the year before their death.

Our conservative assumption is too simplistic; in reality there are re-admissions to hospital, thus the counts of cases do not necessarily equal counts of patients. Therefore, the proportion of people using healthcare and social care services is expected to be lower than the estimates we have computed here. The implication of this is that there is only a small number of asthma patients who have high care needs and for whom public expenditure is very steep. There is thus the potential for risk stratification, using prognostic factor research,^19^ and case management, to reduce the risk of hospitalisations, ICU admissions, near death situations and deaths. Recent work has found that about 2% of patients contribute to about 50% of healthcare costs in Scotland.^15,20^ Although our work alludes towards this fact, given the limitations in the data we could not ascertain this in the asthma patient population. Making such inference will require a cohort study design, which will permit investigating whether there is a HNHC/HRI/HHG asthma patient group in Scotland, and if there is, estimates of proportions of HHG and HC asthma patients.

### Implications for policy, practice and research

This study, through the pyramid structure of the disease portrayal, very clearly demonstrates that Scotland has both a pressing need and the data assets needed to address the issue of identifying HNHC/HRI/HHG patients. Although asthma should in the vast majority of cases be manageable in primary care contexts, our study found that in Scotland—despite the NHS spending around £100 m/year—there are nearly 8000 hospitalisations and 100 deaths from asthma/year. Much of this expenditure and poor outcomes is down to a small percentage of people who are not always easy to identify and manage. Having studied the overall patterns of care and costs of asthma in Scotland,^2^ we now need to build on this and develop a new tool that allows healthcare professionals to find patients at risk of poor asthma outcomes. We plan to do this by analysing Scotland’s unique national data sets, through linkage, to create a cohort of asthma patients to help understand and identify patients who could gain from better case management earlier on, than letting them become severely sick and costly. Once we have found this patient group, we need to find new ways to give them more tailored care, so they do better. Similar work is also needed in other UK and European nations, to understand the asthma population profiles in each of their respective nations, for better care and resource allocation.

## Conclusions

Although asthma is common in Scotland, only a relatively small proportion of those with asthma experience attacks requiring acute hospital attendance/admission in any given year. It is important to characterise the relatively small subset of patients who are experiencing poor asthma outcomes and see if it is possible to develop and validate prognostic algorithms for identifying those with severe asthma attacks in Scotland, and then armed with this information proactively intervene to reduce the risk of severe, potentially life-threatening asthma attacks.

## Methods

We undertook a national cross-sectional study in Scotland. The STROBE and RECORD statements were used to guide the reporting of this manuscript (online Appendix 1).^21,22^

### Data sources used

We undertook secondary analyses of Scottish Health Survey (SHeS) and National Health Service (NHS) data sets on primary and secondary care and administrative data.^23^ SHeS is a series of stratified, cluster-sampled, cross-sectional surveys designed to measure the health of a representative sample of the Scottish population living in private households.^24^ The NHS data sets used were Quality and Outcomes Framework (QOF), Practice Team Information (PTI), NHS 24, Accident and Emergency (A&E) datamart, Scottish Ambulance Service (SAS), Scottish Morbidity Records (SMR) for outpatients (SMR0) and inpatients (SMR1), Paediatric Intensive Care Audit Network (PICANet) and Scottish Intensive Care Society Audit Group (SICSAG).^23^ The administrative data sets used were disability living allowance (DLA) from Department of Work and Pensions (DWP) and death registrations from National Records Scotland (NRS).^23^

All GP practices in Scotland report to QOF, whereby they get financial incentive for providing quality care to their patients, according to the UK General Medical Services (GMS) contract.^25^ PTI is a general practitioner (GP) database comprising a sample of 60 practices representing about 6% of Scottish GP practices and around 6% of the Scottish patient population.^26^ NHS 24 is a national telephone triage and advice service across Scotland during out-of-hour GP service.^27^ NHS 24 calls are triaged by call-handling nurses using disease-specific algorithm to support decision-making. A&E datamart (A&E2) was used for sites, which report patient-level information from their A&E departments.^28^ SAS provides ambulance service across Scotland. All outpatient clinic appointments are recorded in SMR0 and all hospital discharges in SMR1 across Scotland.^29^ PICANet is a UK-wide audit network, which collects data from all paediatric intensive care units (ICU).^30^ SICSAG is a Scotland-wide audit network that captures data from all adult ICUs.^31^ Data from stand-alone HDUs are not included in PICANet or SICSAG. All records of number of people and amount of DLA were available from DWP.^32^ All deaths were registered with NRS.^33^

### Outcome measures

Table 1 below describes the outcome measures by data sets used for asthma population profile in Scotland, with the name of data set, population coverage in the data set and criteria used to select study population from that data set.

**Table 1:** Outcome measures by data sets used for asthma population profile in Scotland—name of data set, population coverage in the data set and criteria used to select study population from that data set

Outcome		Data sets used for Scotland		
		Name of data set	Population coverage	Selection criteria for study population
Prevalence	Annual prevalence of clinician-reported-and-diagnosed asthma	Practice Team Information (PTI)	Sixty GP practices in National Health Service (NHS) Scotland (6% of all GP practices)^a^	Read codes, version 2, for asthma diagnosis^b^
	Annual prevalence of clinician-reported-diagnosed-and-treated asthma	Quality and Outcomes Framework (QOF)	All GP practices in NHS Scotland (about 1000; number changes slightly with time)	Read codes, version 2, for asthma diagnosis^b^
	Lifetime prevalence of patient-reported symptoms suggestive of asthma^b^ Annual prevalence of patient-reported symptoms suggestive of asthma^c^ Lifetime prevalence of patient-reported clinician-diagnosed asthma^d^ Annual prevalence of patient-reported clinician-diagnosed symptomatic asthma^e^ Annual prevalence of patient-reported clinician-diagnosed-and-treated asthma^f^	Scottish Health Survey (SHeS)	National population survey of randomly sampled private household	Answer to survey questions^c–g^
Primary care- healthcare utilisation	GP and nurse consultation	Practice Team Information (PTI)	Sixty Scottish GP practices (6% of all GP practices)^a^	Read codes, version 2, for asthma diagnosis^a^
	Out of hours calls	NHS 24	All telephone calls to NHS 24 in NHS Scotland	Calls triaged using NHS 24’s asthma-specific algorithm
Secondary care— healthcare utilisation	Ambulance conveyances	Scottish Ambulance Service (SAS)	All ambulance conveyances in Scotland	SAS record had ‘Emergency call-asthma selected’
	Accident and emergency (A&E) visits	Accident and Emergency datamart	All hospitals with A&E in NHS Scotland	ICD-10 codes for asthma,^h^ including ‘R062’ (family history of asthma) or if the ‘presenting complaint text’ or ‘diagnosis text’ referred to any of the terms asthma, wheezing, low saturation, chest tightness or shortness of breath
	Inpatient and day-case episodes (non-psychiatric)	Scottish Morbidity Records (SMR01)	All hospitals in NHS Scotland	ICD-10 codes for asthma as primary reason for admission^h^
	Paediatric ICU episodes	Paediatric Intensive Care Audit Network (PICANet)	All paediatric intensive care units in NHS Scotland	Read codes, version 3, as primary reason for admission^i^
	Adult ICU episodes	Scottish Intensive Care Society Audit Group (SICSAG)	All adult intensive care units in NHS Scotland	APACHE III diagnoses for asthma as primary reason for admission
Societal	Disability living allowance	Department for Work and Pensions (DWP)	All individuals claiming benefit in Scotland	ICD-10 codes for asthma as primary reason for admission^h^
	Deaths	National Records of Scotland (NRS)	All individuals who died in Scotland	ICD-10 codes for asthma as underlying cause of death^h^

^a^The total population of PTI GP practhe Scottish population with regards to age, gender and deprivation, and small imbalances due to these demographic and socio-economic factors are addressed during data analysis through a process of direct standardisation

^b^Read codes, version 2 listed: http://bmjopen.bmj.com/content/bmjopen/suppl/2014/11/04/bmjopen-2014-006647.DC1/bmjopen-2014-006647supp_appendix2.pdf

^c^‘Have you ever had wheezing/whistling in the chest at any time, either now/in the past?’

^d^‘Have you had wheezing or whistling in the chest in the last 12 months?’

^e^‘Did a doctor ever tell you that you had asthma?’

^f^‘Have you had wheezing or whistling in the chest in the last 12 months?’ and ‘Did a doctor ever tell you that you had asthma?’

^g^‘Were you treated in the past 12 months for wheeze by GP/nurse at surgery/community/school/district nurse/hospital, consultant/specialist at hospital, consultant/specialist elsewhere, homoeopath/acupuncturist/other alternative medicine professional?’

^h^ICD-10 codes ‘J 45’ (asthma), ‘J46’ (status asthmaticus)

^i^Read codes, version 3 listed: http://bmjopen.bmj.com/content/bmjopen/suppl/2014/11/04/bmjopen-2014-006647.DC1/bmjopen-2014-006647supp_appendix3.pdf

SHeS was used to describe the lifetime and annual prevalence of patient-reported symptoms suggestive of asthma, lifetime prevalence of patient-reported clinician-diagnosed asthma, annual prevalence of patient-reported clinician-diagnosed symptomatic asthma and annual prevalence of patient-reported clinician-diagnosed-and-treated asthma. The relevant questions used to determine these prevalence outcome measures have been previously described.^2^ QOF was used for annual prevalence of clinician-reported-diagnosed-and-treated asthma.^25^ PTI was used for annual prevalence of clinician-reported-and-diagnosed asthma and GP and nurse consultations, by using Read codes version 2.^23,26^ Number of calls for asthma during GP out-of-hours services were obtained from NHS 24.^23,27^ Only ‘new’ and ‘unplanned return’ type of attendances at A&E were used, and not when patients had a scheduled visit to A&E clinics, classified as ‘recall’ or ‘planned return’. Furthermore, A&E visits were selected based on ICD-10 codes ‘J45’ (asthma) and ‘J46’ (status asthmaticus), along with ‘R062’ (family history of asthma) or if the ‘presenting complaint text’ or ‘diagnosis text’ referred to any of the terms or textual variations of: asthma, wheezing, low saturation, chest tightness or shortness of breath.^23,28^ The NHS Health Boards of Orkney and Tayside did not submit patient level information to A&E2, and hence these two regions were excluded from the A&E analysis. The number of ambulance conveyances for asthma were obtained from SAS.^23,34^ SMR0 was not used since diagnoses in outpatient clinics are not recorded.^23,35^ Hospital discharge episodes for asthma were obtained from SMR01, which had ICD-10 codes ‘J45’ and ‘J46’ as the primary diagnosis.^23,35^ ICUs discharges for Scottish residents with primary diagnosis of asthma were obtained from PICANet using Read codes version 3 and from SICSAG using Acute Physiology and Chronic Health Evaluation (APACHE) III diagnoses.^30,31,36^ Paediatric and adult ICU episodes have been added up to generate estimates of ICU episode numbers at population level. The number of people who received DLA for asthma as their main disabling condition were available from DWP,^32^ using ICD-10 codes. The number of deaths with asthma as the underlying cause of death, using ICD-10 codes, were available from NRS.^37^

We had access to anonymised SHeS records at individual respondent level from the open data source from UK Data Services.^24^ QOF was obtained and is available aggregated, at individual GP level. All the other data, except QOF, were available aggregated by 5-year age-group and gender to ensure patient confidentiality by data custodians.

### Study period

Data from healthcare and administrative data sets were obtained for the financial year 2011–12. The corresponding latest SHeS data for that time period was for 2010–11.

### Analysis

Where the data set referred to a sample of the population, e.g., SHeS, PTI or to a sub-section of the population, e.g., A&E, estimates were calculated, and then scaled up to the national population level to determine the number estimates at national level. Asthma attendances in A&E were not only missing from two of the 14 NHS Health Boards, but there were also 390 attendances (4.4%) where age/gender information was missing. Counts of A&E attendances by 5-year age-groups and gender from the remaining NHS Health Boards and their total population were used to extrapolate Scotland level numbers, using Scottish mid-year estimate of population (SMYEP) by age-group and gender. SMYEP in 2010 was 5,262,200 and in 2011 was 5,299,900. A similar scaling up of the SHeS estimates were done at Scotland level by age-group and gender. Thus, 95% confidence intervals (CI) were calculated for estimates from SHeS, PTI and A&E, using Poisson approximation.^23^ Where the data set referred to the entire national population, e.g., hospital admissions, the number of episodes or people were taken from the database. Numbers obtained directly from QOF, NHS 24, SAS, SMR01, PICANet, SICSAG, NRS deaths and DLA are reported here. Percentage of the population is reported with 95% CI. Using a pyramid, graph we constructed a national asthma population profile to describe asthma prevalence, healthcare and societal care utilisation and outcomes, including mortality.

### Ethical approval

Analyses were undertaken using anonymised, aggregated data, for the entire work. The application for ethics was processed through The University of Edinburgh’s Centre for Population Health Sciences Research Ethics Committee; and a self-assessment revealed that no further ethical permissions were required. For patient level data access we obtained approvals from the respective Data Custodians.

### Data availability statement

We had access to data which were

(a) Publicly available online, e.g., Scottish Health Survey (https://discover.ukdataservice.ac.uk/catalogue/?sn=6987)

(b) Aggregated by year, sex and age-group level (e.g., hospital discharge data), which are available only to named researchers and cannot be shared without the approval of relevant data custodians

(c) Individual level data (e.g., primary care data), obtained after ethics approval, available only to named researchers and cannot be shared.

## Electronic supplementary material

Appendix 1 STROBE, RECORD Checklist, Appendix 2
