# Supplementary material for: High health gain patients with asthma: a cross-sectional study analysing national Scottish data sets
Source: NPJ Prim Care Respir Med. 2018 Jul 19;28:27. doi: 10.1038/s41533-018-0094-6 (PMC6053372; doi:10.1038/s41533-018-0094-6)
Supplement: Supplementary file 1 — Appendix 1 STROBE, RECORD Checklist, Appendix 2 [file 41533_2018_94_MOESM1_ESM.pdf]

Appendix 1 The RECORD statement – checklist of items, extended from the STROBE statement

	Item No.	STROBE items	Location in manuscript where items are reported	RECORD items	Location in manuscript where items are reported
**Title and abstract**					
	1	(a) Indicate the study's design with a commonly used term in the title or the abstract (b) Provide in the abstract an informative and balanced summary of what was done and what was found	Title, Pg 1	RECORD 1.1: The type of data used should be specified in the title or abstract. When possible, the name of the databases used should be included.<br><br>RECORD 1.2: If applicable, the geographic region and timeframe within which the study took place should be reported in the title or abstract.<br><br>RECORD 1.3: If linkage between databases was conducted for the study, this should be clearly stated in the title or abstract.	Title, Pg 1<br><br>Title, Pg 1 and or Abstract, pg 2<br><br>NA
**Introduction**					
Background rationale	2	Explain the scientific background and rationale for the investigation being reported	Introduction, pg 3		Introduction, pg 3
Objectives	3	State specific objectives, including any prespecified hypotheses	Introduction, pg 3		Introduction, pg 3
**Methods**					
Study Design	4	Present key elements of study design early in the paper	Title, pg 1 and Methods, pg 8		Title, pg 1 and Methods, pg 8
Setting	5	Describe the setting, locations, and relevant dates, including periods of recruitment, exposure, follow-up, and data collection	Methods, pg 8		Methods, pg 8; Table 1

Participants	6	*(a)* *Cohort study* - Give the eligibility criteria, and the sources and methods of selection of participants. Describe methods of follow-up<br>*Case-control study* - Give the eligibility criteria, and the sources and methods of case ascertainment and control selection. Give the rationale for the choice of cases and controls *Cross-sectional study* - Give the eligibility criteria, and the sources and methods of selection of participants<br><br>*(b)* *Cohort study* - For matched studies, give matching criteria and number of exposed and unexposed<br>*Case-control study* - For matched studies, give matching criteria and the number of controls per case	Methods –<br>Data sources, pgs 8;<br>Outcome measures, pgs 8-9	RECORD 6.1: The methods of study population selection (such as codes or algorithms used to identify subjects) should be listed in detail. If this is not possible, an explanation should be provided.<br><br>RECORD 6.2: Any validation studies of the codes or algorithms used to select the population should be referenced. If validation was conducted for this study and not published elsewhere, detailed methods and results should be provided.<br><br>RECORD 6.3: If the study involved linkage of databases, consider use of a flow diagram or other graphical display to demonstrate the data linkage process, including the number of individuals with linked data at each stage.	Methods –<br>Data sources, pgs 8;<br>Outcome measures, pgs 8-9 ;<br>Table 1<br><br>Methods –<br>Data sources, pgs 8;<br>Outcome measures, pgs 8-9<br><br>NA
Variables	7	Clearly define all outcomes, exposures, predictors, potential confounders, and effect modifiers. Give diagnostic criteria, if applicable.	Methods –<br>Outcome measures, pgs 8-9	RECORD 7.1: A complete list of codes and algorithms used to classify exposures, outcomes, confounders, and effect modifiers should be provided. If these cannot be reported, an explanation should be provided.	Methods –<br>Outcome measures, pgs 8-9
Data sources/ measurement	8	For each variable of interest, give sources of data and details of methods of assessment (measurement).<br>Describe comparability of assessment methods if there is more than one group	Methods –<br>Data sources, pgs 8;<br>Outcome measures, pgs 8-9 ;<br>Analysis pg 10		

Bias	9	Describe any efforts to address potential sources of bias	Methods – Analysis pg 10		
Study size	10	Explain how the study size was arrived at	Methods – Analysis pg 10		
Quantitative variables	11	Explain how quantitative variables were handled in the analyses. If applicable, describe which groupings were chosen, and why	Methods – Analysis pg 10		
Statistical methods	12	(a) Describe all statistical methods, including those used to control for confounding (b) Describe any methods used to examine subgroups and interactions<br>(c) Explain how missing data were addressed<br>(d) *Cohort study* - If applicable, explain how loss to follow-up was addressed<br>*Case-control study* - If applicable, explain how matching of cases and controls was addressed<br>*Cross-sectional study* - If applicable, describe analytical methods taking account of sampling strategy<br>(e) Describe any sensitivity analyses	Methods – Analysis pg 10		
Data access and cleaning methods		..		RECORD 12.1: Authors should describe the extent to which the investigators had access to the database population used to create the study population.	Methods – Outcome measures, pgs 8-9 ;<br>Ethical approval, pg 10

				RECORD 12.2: Authors should provide information on the data cleaning methods used in the study.	Methods – Analysis, pg 5-6
Linkage		..		RECORD 12.3: State whether the study included person-level, institutional-level, or other data linkage across two or more databases. The methods of linkage and methods of linkage quality evaluation should be provided.	NA
**Results**					
Participants	13	(a) Report the numbers of individuals at each stage of the study (*e.g.*, numbers potentially eligible, examined for eligibility, confirmed eligible, included in the study, completing follow-up, and analysed)<br>(b) Give reasons for nonparticipation at each stage. (c) Consider use of a flow diagram	Results - Prevalence, pg 3; Healthcare and societal care utilisation, pg 3-4; Fig 1	RECORD 13.1: Describe in detail the selection of the persons included in the study (*i.e.,* study population selection) including filtering based on data quality, data availability and linkage. The selection of included persons can be described in the text and/or by means of the study flow diagram.	Results - Prevalence, pg 3; Healthcare and societal care utilisation, pg 3-4; Fig 1
Descriptive data	14	(a) Give characteristics of study participants (*e.g.*, demographic, clinical, social) and information on exposures and potential confounders<br>(b) Indicate the number of participants with missing data for each variable of interest (c) *Cohort study* - summarise follow-up time (*e.g.*, average and total amount)	Results - Prevalence, pg 3; Healthcare and societal care utilisation, pg 3-4;		Results - Prevalence, pg 3; Healthcare and societal care utilisation, pg 3-4;

Outcome data	15	*Cohort study* - Report numbers of outcome events or summary measures over time<br>*Case-control study* - Report numbers in each exposure			
		category, or summary measures of exposure<br>*Cross-sectional study* - Report numbers of outcome events or summary measures	Results - Prevalence, pg 3; Healthcare and societal care utilisation, pg 3-4;		
Main results	16	(a) Give unadjusted estimates and, if applicable, confounder adjusted estimates and their precision (e.g., 95% confidence interval). Make clear which confounders were adjusted for and why they were included (b) Report category boundaries when continuous variables were categorized<br>(c) If relevant, consider translating estimates of relative risk into absolute risk for a meaningful time period	Results - Prevalence, pg 3; Healthcare and societal care utilisation, pg 3-4;		
Other analyses	17	Report other analyses done—e.g., analyses of subgroups and interactions, and sensitivity analyses			
**Discussion**					
Key results	18	Summarise key results with reference to study objectives	Discussion – Statement of principal findings, pg 9		

Limitations	19	Discuss limitations of the study, taking into account sources of potential bias or imprecision. Discuss both direction and magnitude of any potential bias	Discussion – Limitations, pg 5; Interpretation in the light of the previous literature, pg 5-7	RECORD 19.1: Discuss the implications of using data that were not created or collected to answer the specific research question(s). Include discussion of misclassification bias, unmeasured confounding, missing data, and changing eligibility over time, as they pertain to the study being reported.	
Interpretation	20	Give a cautious overall interpretation of results considering objectives,	Discussion, pgs 4-7		
		limitations, multiplicity of analyses, results from similar studies, and other relevant evidence	Discussion – Limitations, pg 5; Interpretation in the light of the previous literature, pg 5-7		
Generalisability	21	Discuss the generalisability (external validity) of the study results	Discussion – Interpretation in the light of the previous literature, pg 5-7		
**Other Information**					
Funding	22	Give the source of funding and the role of the funders for the present study and, if applicable, for the original study on which the present article is based	Funding, pg 12		
Accessibility of protocol, raw data, and programming code		..		RECORD 22.1: Authors should provide information on how to access any supplemental information such as the study protocol, raw data, or programming code.	Methods; Data Availability Statement, pg 11; Reference number 23

*Reference: Benchimol EI, Smeeth L, Guttmann A, Harron K, Moher D, Petersen I, Sørensen HT, von Elm E, Langan SM, the RECORD Working Committee. The REporting of studies Conducted using Observational Routinely-collected health Data (RECORD) Statement. *PLoS Medicine* 2015; in press.

*Checklist is protected under Creative Commons Attribution (CC BY) license.

Appendix 2: Population distribution by gender in European Standard Population (ESP) 2013 and Scottish mid-year estimate of population (SMYEP) 2011

Age group	ESP	MYE	MYE females	MYE males
0-4	5,000	293,586	143,840	149,746
5-9	5,500	270,900	132,307	138,593
10-14	5,500	290,266	141,500	148,766
15-19	5,500	326,831	160,460	166,371
20-24	6,000	365,580	183,485	182,095
25-29	6,000	346,349	175,957	170,392
30-34	6,500	323,786	164,142	159,644
35-39	7,000	336,101	171,632	164,469
40-44	7,000	393,664	202,791	190,873
45-49	7,000	410,769	210,561	200,208
50-54	7,000	377,317	192,620	184,697
55-59	6,500	331,924	169,164	162,760
60-64	6,000	336,463	171,835	164,628
65-69	5,500	264,413	137,998	126,415
70-74	5,000	220,367	119,703	100,664
75+	9,000	411,584	251,605	159,979
All ages	100,000	5,299,900	2,729,600	2,570,300
